# Maternal depression during the perinatal period and its relationship with emotion regulation in young adulthood: An fMRI study in a prenatal birth cohort

**DOI:** 10.1017/S0033291725000042

**Published:** 2025-02-12

**Authors:** Klara Mareckova, Filip Trbusek, Radek Marecek, Jan Chladek, Zuzana Koscova, Filip Plesinger, Lenka Andrysková, Milan Brazdil, Yuliya S. Nikolova

**Affiliations:** 1 Brain and Mind Research, Central European Institute of Technology (CEITEC), Masaryk University, Brno, Czech Republic; 2 First Department of Neurology, Faculty of Medicine, Masaryk University and St. Anne’s University Hospital, Brno, Czech Republic; 3 Institute of Scientific Instruments, The Czech Academy of Sciences, Brno, Czech Republic; 4 RECETOX, Faculty of Science, Masaryk University, Brno, Czech Republic; 5 Centre for Addiction and Mental Health (CAMH), University of Toronto, Toronto, ON, Canada; 6 Department of Psychiatry, University of Toronto

**Keywords:** emotion regulation, fMRI, heart rate variability, maternal perinatal depression, prenatal birth cohort, skin conductance

## Abstract

**Background:**

Maternal perinatal mental health is essential for optimal brain development and mental health of the offspring. We evaluated whether maternal depression during the perinatal period and early life of the offspring might be selectively associated with altered brain function during emotion regulation and whether those may further correlate with physiological responses and the typical use of emotion regulation strategies.

**Methods:**

Participants included 163 young adults (49% female, 28–30 years) from the ELSPAC prenatal birth cohort who took part in its neuroimaging follow-up and had complete mental health data from the perinatal period and early life. Maternal depressive symptoms were measured mid-pregnancy, 2 weeks, 6 months, and 18 months after birth. Regulation of negative affect was studied using functional magnetic resonance imaging, concurrent skin conductance response (SCR) and heart rate variability (HRV), and assessment of typical emotion regulation strategy.

**Results:**

Maternal depression 2 weeks after birth interacted with sex and showed a relationship with greater brain response during emotion regulation in a right frontal cluster in women. Moreover, this brain response mediated the relationship between greater maternal depression 2 weeks after birth and greater suppression of emotions in young adult women (ab = 0.11, SE = 0.05, 95% CI [0.016; 0.226]). The altered brain response during emotion regulation and the typical emotion regulation strategy were also as sociated with SCR and HRV.

**Conclusions:**

These findings suggest that maternal depression 2 weeks after birth predisposes female offspring to maladaptive emotion regulation skills and particularly to emotion suppression in young adulthood.

## Introduction

Emotion regulation is an important part of daily life and is necessary for adequate social functioning and overall mental health of individuals (McRae & Gross, 2020). Cognitive reappraisal and emotion suppression have been described as two main strategies of emotion regulation (Gross & Barrett, 2011; Hu et al., 2014; Morawetz et al., 2017). The use of cognitive reappraisal is a more effective strategy in regulating emotional outcomes (Webb et al., 2012) and has been associated with better overall mental health outcomes than suppression of emotional expression (Hu et al., 2014; John & Gross, 2004). Difficulties in emotion regulation are common across various mental disorders (Sheppes et al., 2015), including affective disorders such as depression and anxiety disorders (Aldao et al., 2010), and spanning also neurodevelopmental conditions such as Attention Deficit Hyperactivity Disorder (ADHD) (Shaw et al., 2014) and autism spectrum disorder (Mazefsky et al., 2013). According to Li et al (Li et al., 2019), one’s emotion regulation and lability is associated with parental emotion dysregulation and this intergenerational effect is transmitted indirectly through parental reactions to children’s negative emotions. Recent research in almost 2000 adolescents (Maruyama et al., 2023) then linked worse self-esteem and emotion regulation problems in adolescents with maternal depression.

Emotion regulation is supported by a relatively well-characterized neural circuitry involving frontoparietal and subcortical regions. According to a meta-analysis by Frank et al. (Frank et al., 2014), successful emotion regulation is associated with decreased response in the amygdala, parahippocampal cortex, and inferior parietal lobe (IPL), and increased response in superior, middle, and inferior frontal gyrus, supplementary motor area (SMA), left middle temporal and angular gyrus, and left anterior cingulate cortex. Morawetz et al. (Morawetz et al., 2017) studied different emotion regulation strategies and reported that while reappraisal strategies were associated with greater response of superior and inferior frontal gyrus, dorsomedial prefrontal cortex, superior and middle temporal gyri, IPL, SMA, and preSMA, emotion suppression was associated with converging response in the right IPL and left inferior frontal gyrus. Consistently, research from our group (Mareckova et al., 2016) eliciting negative affect using negative images from the International Affective Picture System showed a greater inability to regulate the amygdala as mood became more dysphoric. Research in patients with major depressive disorder (MDD) versus healthy controls then concluded that MDD patients display abnormalities in higher-level cognitive as well as subcortical limbic structures, which manifest as an inability to appropriately regulate one’s emotions (Park et al., 2019). Diminished ability to downregulate emotions in MDD was also linked with lower activity of lateral prefrontal cortex (Ebneabbasi et al., 2021).

Better emotion regulation has also been associated with greater heart rate variability (Appelhans & Luecken, 2006; Park & Thayer, 2014), which is defined as the variation in time between the heartbeats. The autonomic nervous system (ANS), which influences the heart rate, consists of the sympathetic (SNS) and parasympathetic (PNS) nervous system. While the SNS predominates during stress and is related to an increase in heart rate and skin conductivity, the PNS is the “relaxed response” system, which predominates during the quiet and relaxing states and is related to a decrease in heart rate and skin conductance (Zaehringer et al., 2020). Heart rate variability (HRV) has been associated with emotional arousal and anxiety (Jonsson, 2007; Nickel & Nachreiner, 2003) and it has been demonstrated that HRV can be modulated by the prefrontal cortex (Napadow et al., 2008). In contrast, patients with major depressive disorder (MDD) are known to have lower levels of skin conductivity (Iacono & Lykken, 1983; Kim et al., 2019) and higher high-frequency power (HFn) during HRV measurements (An et al., 2020) than healthy controls.

According to the theory of prenatal programming (Cao-Lei et al., 2020; Kim et al., 2015), the uteroplacental environment of the developing fetus has a long-lasting impact on the offspring. Longitudinal data from several birth cohort studies showed that prenatal maternal anxiety and/or depression were associated with a higher risk for emotional and behavioral problems in childhood (consistent across measurements from 4 to 13 years) and clinical levels of anxiety and depression in adulthood (Acosta et al., 2019; Monk et al., 2019; O’Donnell et al., 2014; O’Donnell & Meaney, 2017). Moreover, these effects could not be explained by maternal prenatal smoking and substance use, birth weight and gestational age of the child, postpartum symptoms of anxiety or depression, maternal parenting, or social circumstances such as maternal education and socioeconomic status (O’Donnell et al., 2014). Recent research also linked perinatal maternal symptoms of depression and anxiety with self-regulation (Schwarze et al., 2024) and emotion regulation (Amani et al., 2024) in their infants. Within the current project, we focused on the effects of maternal depression during the perinatal period and early life of the offspring on emotion regulation skills in young adult offspring.

Numerous studies pointed out the importance of the timing of the exposure during the perinatal period (Mareckova et al., 2020; Teicher et al., 2006), because different brain regions have unique periods when they are maximally sensitive to the effects of early adversity. Based on this literature, we hypothesized that perinatal maternal depression and particularly depression during the prenatal period will be associated with altered brain function during emotion regulation and manifest as worse emotion regulation skills in young adulthood. Given previous research, which demonstrated greater vulnerability of women to affective disorders (Nolen-Hoeksema, 2012; Seedat et al., 2009), sex differences in emotion regulation and psychopathology (Nolen-Hoeksema, 2012), and sex differences in brain response during emotion regulation (McRae et al., 2008), we hypothesized that these effects might be particularly pronounced in women. We also hypothesized that the altered brain function during emotion regulation as well as the typical use of specific emotion regulation strategies will be reflected by altered skin conductance and heart rate variability during emotion regulation. Finally, we hypothesized that the altered brain response during emotion regulation will mediate the relationship between maternal depression and worse emotion regulation skills.

## Methods and materials

### Participants

We studied young adults from the Czech Republic who participated in the European Longitudinal Study of Pregnancy and Childhood (ELSPAC) (Piler et al., 2017) and its neuroimaging follow-up study *Health Brain Age* (HBA). The ELSPAC study comprises a prenatal cohort consisting of 5151 individuals, whose members were born between 1991 and 1992. The initial ELSPAC questionnaires were administered to the mothers upon enrolment into the study at the 20th week of pregnancy, while the final ELSPAC questionnaire was completed by the child at the age of 19. The HBA neuroimaging follow-up was conducted in a subgroup of the ‘ELSPAC children’ (n = 262, 49% females, all of White European ancestry, as indicated by self-report questionnaire) at the age of 28–30 years. All participants provided written informed consent to participate in the HBA study, including the agreement to merge data from HBA with their historical data from ELSPAC. Ethical approval for the HBA study was obtained from the ELSPAC ethics committee. However, only a total of 163 participants (49% females; see Demographics Table in Supplementary Table 1) had maternal depression data from all 4 timepoints during the perinatal and early life period as well as fMRI data at the age of 28 to 30 years and thus could have been included in the current study.

### Procedures

#### Maternal depression during the perinatal period and early life of the offspring

Maternal depression was assessed using the Edinburgh Postnatal Depression Scale (EPDS) (Cox et al., 1987; Gibson et al., 2009) at 4 time points during the perinatal period and early life of the offspring: (a) mid-pregnancy (approx. Week 20), (b) 2 weeks after birth, (c) 6 months after birth, and (d) 18 months after birth.

#### Acquisition and analysis of MRI and fMRI data in young adulthood

Regulation of negative affect in the offspring was studied using the International Affective Picture System (IAPS) fMRI task. Participants observed negative and neutral images from the IAPS database (Bradley & Lang, 2017), which were selected based on ratings of evoked affect. A total of 66 images were presented to probands during the measurement, 1 image per trial. These 66 trials were divided into 3 conditions (22 images each) – neutral observe, negative observe, and negative regulate. In each trial, the image exposure lasted for the first 8 seconds. The trial started by displaying an image and text “Observe image” for 3 seconds. After 3 seconds the text changed either to “Keep observing the image” (neutral/negative observation trial) or “Regulate the emotions evoked by the image” (negative regulate trial) while the image was still displayed for another 5 seconds. Therefore, the affective response to negative pictures was regulated between the 3rd and 8th second. The trial continued by the display of a blank screen for 1 second and another screen for 3 seconds that asked for a rating by the query “How uncomfortable do you feel right now?” and a scale “not at all – a little – quite a bit – very much.” The trial ends with observing the text “Relax” for 9 seconds to prevent carry-over effects and set up the baseline for neural as well as heart rate and electrodermal activity. Overall, the trial duration was 21 seconds and the complete task took 23 minutes. The order of the trials was pseudo-random, creating 12 variations of the task. The order was set to avoid two trials from the same condition appearing consecutively. Further, the first three trials contained all three conditions in random order. This was the same for the second three trials and all ensuing threes of trials. During the neutral and negative observation conditions, participants were instructed to observe the images. During the negative regulate condition, participants were asked to regulate their affective response.

Magnetic resonance imaging (MRI) of the brain was conducted using a 3 T Siemens Prisma MRI scanner. T1-weighted (T1w) whole-brain MPRAGE images were acquired using 64 channel head/neck coil with acquisition parameters: voxel size 1 mm3, repetition time (TR) 2,300 ms, echo time (TE) 2.34 ms, inversion time (TI) 900 ms, flip angle 8°. Functional MRI images were acquired using the following acquisition parameters: voxel size: 3 × 3 × 3 mm; whole-brain coverage, repetition Time: 0.7 s, FlipAngle: 47°, Effective Echo Spacing: 0.000285002 s, Phase Encoding Direction: “y-,” Multiband Acceleration Factor: 6, Parallel Reduction Factor In Plane: 2, Total slices: 2,121, EchoTime: 0.0164 s (Echo1); 0.03766 s, (Echo 2); 0.05892 s (Echo 3). For each measurement, 3 frames were captured with different Echo time parameters, which were subsequently combined into 1 frame as part of the data preprocessing.

The fMRI data were processed using SPM12 (https://www.fil.ion.ucl.ac.uk/spm/software/spm12/) toolbox running under MATLAB (The MathWorks, Inc.). The preprocessing comprised steps as follows: All echo 2 data were realigned and resliced and echo 1 and 3 data were resliced using parameters from echo 2 data. Voxel displacement map was estimated according to the fieldmap data and applied to correct data from echoes 1, 2, and 3. Next, the 3 echo datasets were merged using temporal SNR weighting approach. Subsequently, the mean fMRI image was registered to the structural image, which was spatially normalized to the MNI152 template. Data were smoothed using FWHM of 5 mm and all pre-processed data were checked for subject motion. One subject was excluded due to more than 20% of scans with a framewise displacement of 0.5 mm (Power et al., 2014; Power et al., 2015; Pupikova et al., 2022). There was no difference in framewise displacement between men and women (Supplementary Figure 1).

Next, a whole-brain voxel-wise analysis used a general linear model (GLM) that was estimated for each subject. The GLM design matrix contained 3 regressors for each condition: a regressor that modelled initial phase of a trial (“observe image”) another for the second phase of the trial (“keep observing” or “regulate”) and another for the last phase of the trial (the rating). Each condition was modeled as an boxcar function convolved with canonical hemodynamic response function provided by SPM12 (HRF). The GLM was amended by additional regressors that modeled (a) low frequency fluctuations (a cutt-off frequency of 1/128 s), (b) signal variability caused by subject’s movements (transitions, rotations and their differencies) and (c) neural activity that corresponded to execution of key strokes (a brief events convolved with HRF). These additional regressors effectively filtered nuisance variability comprised in the data (e.g. physiological noise and neural activity linked to response execution). After GLM estimation we set the following contrasts based on effects of the regressors that modelled the second phase of the trials: (1) negative observe > neutral observe and (2) negative regulate > negative observe. These contrasts estimates entered the group-level statistics.

#### Acquisition and analysis of skin conductance data in young adulthood

Skin conductance response (SCR) data were collected during the fMRI task using the ExG MR compatible system – BrainProducts BrainAmp ExG MR, allowing SCR data to be calculated for the negative observe versus neutral observe as well as the negative regulate versus negative observe contrasts. Similarly to the fMRI data analysis, the SCR data analysis focused on the 5-second window when participants saw the negative or neutral image with instructions to either simply observe or regulate the emotion it elicits in them.

First, EDA signal quality was assessed based on the recommendations from previous research (Benedek & Kaernbach, 2010; Boucsein, 2012). A semi-automatic classifier included the following steps: (1) An independent expert evaluator (JCH) excluded data with incomplete measurements and easily visible artifacts – e.g. noisy data with no visible signal, signals with abrupt level changes; (2) An automatic artifact detector “ft_artifact_jump” implemented in the Fieldtrip Toolbox (Oostenveld et al., 2011) was used to detect “jumps” in the data stream based on z-scores and data containing these “jump’ artifacts were excluded from further analysis, except for isolated spikes, which were removed using LedaLab Toolbox; (3) Since the lack of contact between an electrode and the skin results in a fall of the EDA signal to a minimal constant value (Böttcher et al., 2022; Nasseri et al., 2020), flat segments in the EDA data were identified by an expert evaluator (JCH) and data with more than 50% of flat record were excluded from further analysis. This lack of contact between an electrode and the skin was the most common reason for data exclusion (cca 75% of the excluded data).

Preprocessing of the SCR data was performed using Brain Vision Analyzer 2.1 software (Brain Products GmbH, Germany) to suppress MR gradient artifacts. Next, data were filtered by a low-pass Finite Impulse Response (FIR) filter with a cut-off frequency 40 Hz and downsampled to 250 Hz. The FIR filter was a Raised-Cosine filter (beta = 1) designed by the windowing method using a Hanning window. Further preprocessing was done using the LedaLab v3.4.9, a MATLAB Toolbox (Benedek & Kaernbach, 2010). SCR data were low-pass filtered, and resampled to 25 Hz, the remaining artifacts were corrected by adaptive smoothing with a Gaussian window and subsequently decomposed into its tonic and phasic components using a Continuous Decomposition Analysis (CDA) strategy. The number of optimization initial sets was in the range of 2–4. Next, the event-related sympathetic activity was analyzed relative to stimulus onset while the minimum amplitude criterion used was 0.01 muS. Next, standard trough-to-peak (TTP) analysis was used to extract the number of significant SCR within the response window (nSCR), the response latency of the first significant SCR within the response window (latency), and the sum of the SCR amplitudes within the response window (sum of amplitudes). Good quality skin conductance data (e.g. data with a complete set of trials, without a flat curve or technical artifacts due to movement or bad electrode contact) were available for 144 participants (57 women, 137 men) with ERQ data and 86 of these participants (37 women, 49 men) had also complete data on maternal depression during the perinatal period and early life of the offspring and fMRI data.

#### Acquisition and analysis of HRV in young adulthood

During the fMRI task, the electrocardiogram (ECG) signals were collected and, subsequently, interbeat (RR) intervals were extracted. Next, the freely available Python package “hrv-analysis” (https://pypi.org/project/hrv-analysis/) was used to pre-process RR intervals and extract HRV features. Briefly, the data underwent outlier removal, interpolation of missing values, and ectopic beat removal, ensuring the integrity of the signal for further HRV analysis. Finally, time-domain HRV features were extracted for the negative observe versus neutral observe as well as the negative regulate versus negative observe contrasts. We focused on the high-frequency band (HF; 0.15–0.50 Hz), which reflects the parasympathetic modulation, and calculated the normalized high-frequency bands (HFn), expressed as percentages and calculated as the absolute power area of the high-frequency band divided by the sum of absolute power areas of both low and high-frequency bands multiplied by 100. Good quality HRV data were available for 243 participants (114 women, 129 men) with ERQ data and 155 of these participants (75 women, 80 men) had also complete data on maternal depression during the perinatal period and early life of the offspring and fMRI data.

#### Emotion regulation questionnaire in young adulthood

The Emotion Regulation Questionnaire (ERQ) (Gross & John, 2003) was used to assess participants’ typical emotion regulation strategy. The ERQ questionnaire includes 10 questions on a 7-point Likert scale to evaluate (1) one’s ability to cognitively reappraise emotions and thus being able to change the actual emotional experience when regulating emotions (e.g. “When I want to feel a less negative emotion such as sadness or anger, I change what I’m thinking about.”) and to evaluate (2) one’s ability of expressive suppression and thus being able to suppress the emotions in their talk, gestures or behavior (e.g. “When I want to feel less negative emotions, I make sure not to express them”). This self-assessment questionnaire was done on the same day as the MRI session.

### Statistical analyses

First, we conducted a voxelwise analysis using multiple regression in SPM12 to assess the impact of maternal depression during the perinatal period and early life of the offspring on brain response during emotion regulation (the negative regulate versus negative observe contrast). The multiple regression included the 4 EPDS measurements (mid-pregnancy, 2 weeks after birth, 6 months after birth, 18 months after birth) and the interaction with sex was added as a covariate for each contrast. The level of statistical significance for this voxelwise analysis was set to p < 0.05 and was corrected for multiple comparisons using the Family-Wise Error (FWE) correction. Next, we used a general linear model in JMP version 10.0.0 (SAS Institute Inc., Cary, NC) to test the relationships between the maternal depression-associated brain response during emotion regulation and the typical use of emotion regulation strategy (cognitive reappraisal, emotion suppression). Multiple comparisons were corrected using the False Discovery Rate (FDR) method. Finally, we used SPSS version 27 to conduct a mediation analysis to determine whether the brain response during emotion regulation, which showed a significant relationship with emotion regulation skills (the use of cognitive reappraisal and emotion suppression strategies), might mediate the relationship between maternal depression and the emotion regulation skills in young adulthood. The significance of the indirect effect (Preacher & Hayes, 2004) was determined using a bootstrap procedure. A total of 5,000 bootstrap resamples were used to provide stable estimates of the direct, indirect, and total effects. We determined 95% confidence intervals from the bootstrap resamples and any interval that did not include 0 was considered significantly different from 0. All these analyses were conducted in n = 163.

Subsequently, a general linear model in JMP version 10.0.0 (SAS Institute Inc., Cary, NC) evaluated the relationship of the maternal depression-related brain response during the emotion regulation task, which showed a significant relationship with emotion regulation skills, and (1) skin conductance (n = 86) and (2) heart rate variability (n = 155). Finally, we assessed the potential relationship of skin conductance (n = 144) and heart rate variability (n = 243) with the typical use of emotion regulation skills in young adulthood. Sex was considered as a potential moderator. Multiple comparisons were corrected using the FDR method within each family of analyses.

## Results

Information on the range of maternal depression at the 4 different time points during the perinatal period and early life of the offspring is available in Supplementary Table 2. As detailed in Supplementary Table 3, the Cronbach alpha values were > 0.7, indicating internal consistency and reliability of the measures. The correlations between the different measures are provided in Supplementary Table 4. The variance inflation factors (VIF) for these relationships (VIF = <1), indicating no multicollinearity issues.

Patterns of brain response to the 2 contrasts of interest – (1) Negative observe versus neutral observe and (2) Negative regulate versus negative observe are also presented in the Supplementary Materials.

### Maternal depression during the perinatal period and early life of the offspring and brain function during emotion regulation in young adulthood

**Figure 1.**
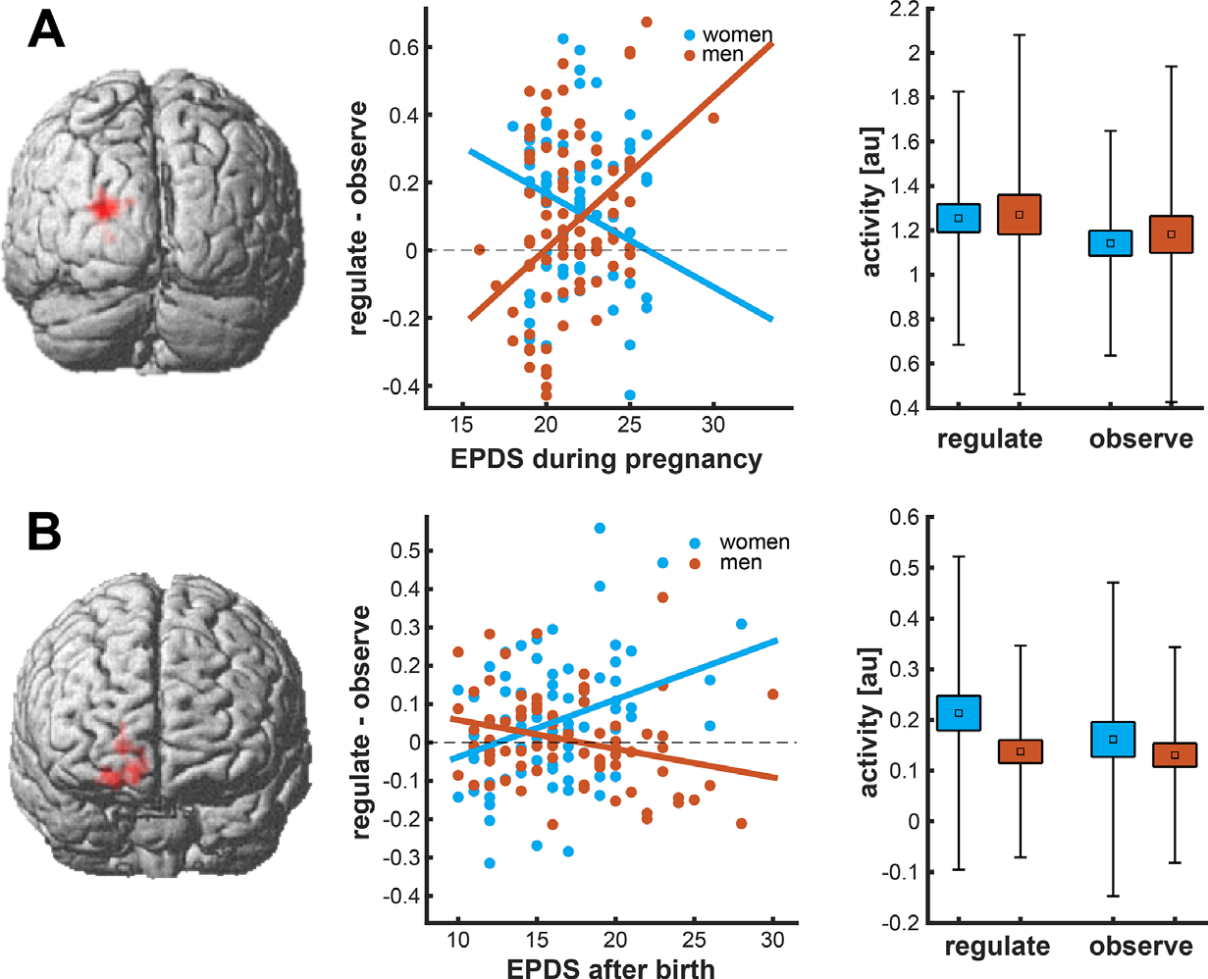
Maternal mental health during the perinatal period and brain response during emotion regulation task in young adulthood. Greater maternal depression during pregnancy was associated with lower brain response in the left occipital cluster in women (blue) during the regulate vs observe negative contrast (1A). Greater maternal depression after birth was associated with greater brain response in the right frontal cluster in women (blue) during the regulate vs observe negative contrast (1B). The box represents the standard error, the whiskers represent the standard deviation, and the square represents the mean activity.

Multiple regression showed two statistically significant interactions between maternal depression and offspring sex on brain response during the negative regulate versus negative observe contrast. In women (but not men), maternal depression in mid-pregnancy was associated with lower brain response in one cluster of voxels including the left middle occipital gyrus, cuneus, and occipital superior gyrus (68 voxels, peak T = 4.33, MNI coordinates x = −24, y = −91, z = 17, cluster FWEp = 0.001; see Figure 1a). In women (but not men), maternal depression 2 weeks after birth was associated with greater brain response in a cluster including superior frontal gyrus and anterior orbitofrontal cortex (11 voxels, peak T = 4.50, MNI coordinates x = 12, y = −71, z = 10, cluster FWEp = 0.004; see Figure 1b).

We did not find any statistically significant evidence of an association of maternal depression during the perinatal period and early life of the offspring and brain activity during the condition observe negative > neutral.

### Maternal depression-related brain response during emotion regulation fMRI task in young adult women and its relationship with typical emotion regulation strategy

Greater brain response in the right frontal cluster associated in young adult women with maternal depression 2 weeks after birth predicted greater use of emotion suppression (beta = 0.27, p = 0.006, FDRp = 0.02, R^2^ = 0.07) but not emotion reappraisal (beta = −0.14, p = 0.22, FDRp = 0.22). There was no relationship between the brain response in the occipital cluster associated in young adult women with maternal depression during pregnancy and emotion suppression (beta = 0.19, p = 0.09, FDRp = 0.15) or emotion reappraisal (beta = −0.18, p = 0.11, FDRp = 0.15).

### Does brain response in the right frontal cluster mediate the relationship between maternal depression 2 weeks after birth and the typical emotion regulation strategy in young adult women?

**Figure 2.**
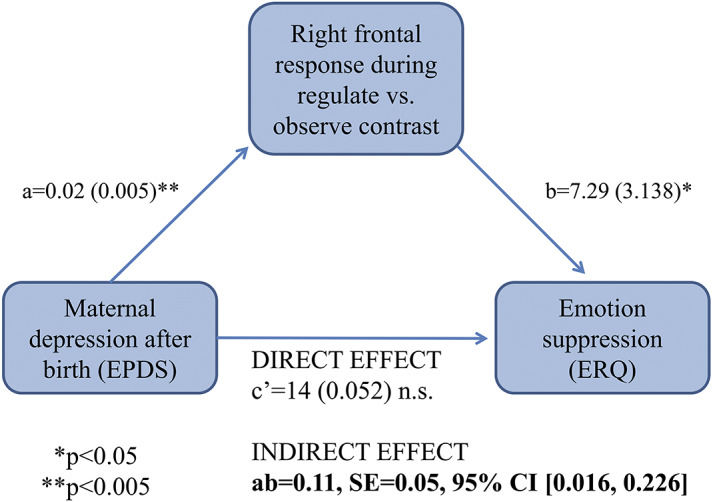
Brain response in the right frontal cluster mediated the relationship between greater maternal depression after birth and greater suppression of emotions in young adult women (ab = 0.11, SE = 0.05, 95% CI [0.016; 0.226]). The a path coefficient refers to the effect of maternal depression after birth on brain response in the right frontal cluster during emotion regulation in young adulthood. The b path coefficient refers to the effect of brain response in the right frontal cluster during emotion regulation on emotion suppression in young adulthood. The indirect effect of maternal depression after birth on emotion suppression in young adulthood through the brain response in the right frontal cluster during emotion regulation in young adulthood is obtained by multiplying a and b path coefficients. The c’ path coefficient refers to the direct effect of the maternal depression after birth on the emotion suppression in young adulthood. Unstandardized regression weights are provided and followed by standard errors (in brackets). n.s. means not significant.

Brain response in the right frontal cluster mediated the relationship between greater maternal depression 2 weeks after birth and greater suppression of emotions in young adulthood (ab = 0.11, SE = 0.05, 95% CI [0.016; 0.226]; see Figure 2).

### Skin conductance and its relationship with brain response during emotion regulation in young adult women

**Figure 3.**
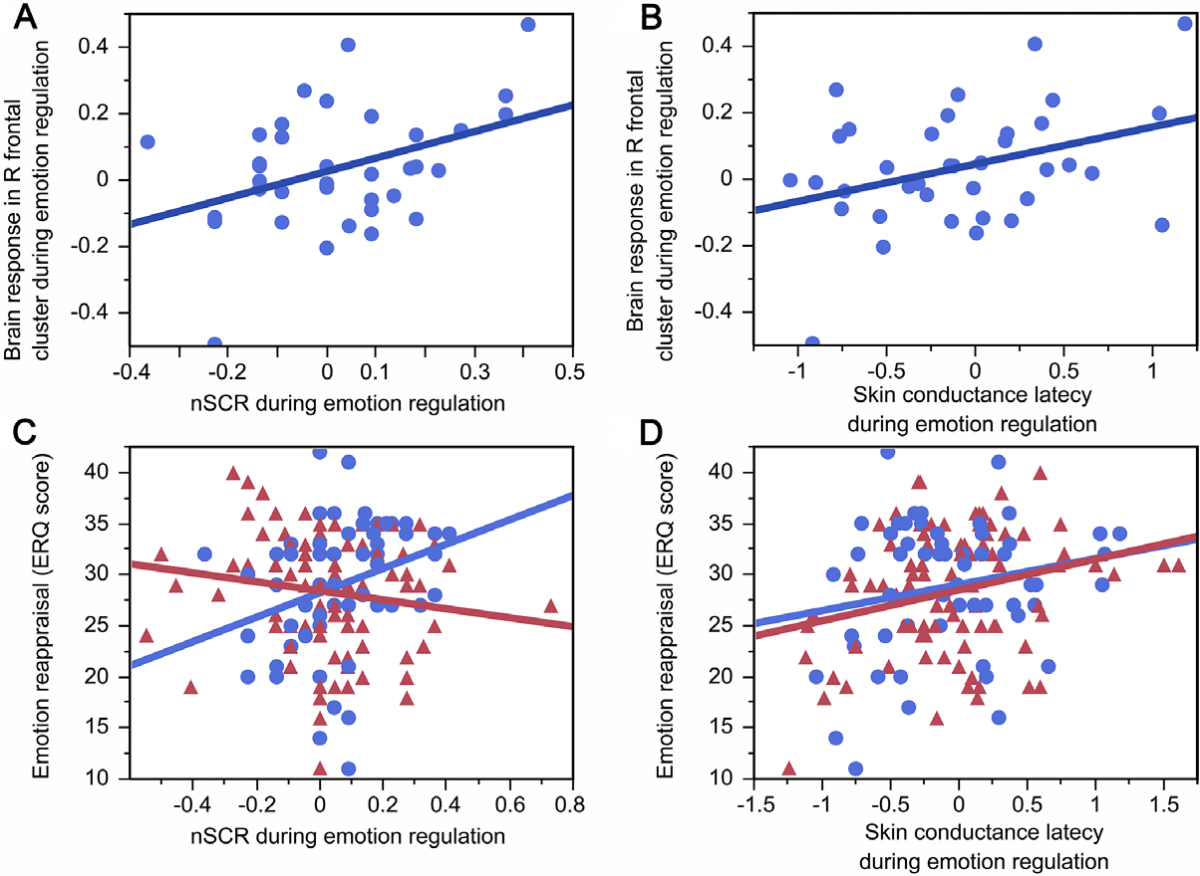
Skin conductance and emotion regulation in young adulthood. Greater nSCR (3A; R^2^ = 0.16, FDRp = 0.01) and greater skin conductance latency (3B; R^2^ = 0.13, FDRp = 0.04) during emotion regulation in women was associated with greater brain response in the right frontal cluster associated with maternal depression after birth. Further, greater nSCR during emotion regulation in women (3C; R^2^ = 0.10, p = 0.02) and greater skin conductance latency during emotion regulation irrespective of sex (3D; AdjR^2^ = 0.04, FDRp = 0.02) predicted more emotion reappraisal. Women are depicted in blue, men are depicted in red.

In young adult women, the greater brain response in the right frontal cluster previously linked with maternal depression 2 weeks after birth was associated with greater nSCR (beta = 0.39, FDRp = 0.01, R^2^ = 0.16; Figure 3A) and greater skin conductance latency (beta = 0.36, FDRp = 0.04, R^2^ = 0.13; Figure 3B), but not greater sum of amplitudes (beta = 0.13, p = 0.44) during the emotion regulation.

### Skin conductance and its relationship with typical emotion regulation strategy

Sex interacted with nSCR (AdjR^2^ = 0.04, beta = 0.25, FDRp = 0.02; Figure 3c) and predicted greater emotion reappraisal. Posthoc analyses revealed that the relationship between greater nSCR during emotion regulation and more emotion reappraisal was significant in women (beta = 0.31, p = 0.02, R^2^ = 0.10) but not men (beta = −0.15, p = 0.16). In addition, greater skin conductance latency predicted greater emotion reappraisal irrespective of sex (AdjR^2^ = 0.04, beta = 0.24, FDRp = 0.02; Figure 3d). No relationship appeared with the sum of amplitudes (beta = 0.05, FDRp = 0.69). Also, no similar relationships appeared with emotion suppression (p > 0.19).

### Heart rate and its relationship with brain response during emotion regulation in young adult women

There was no relationship between the HFn during emotion regulation and brain response in the right frontal cluster associated with maternal depression 2 weeks after birth in young adult women (beta = 0.11, p = 0.36).

### Heart rate and its relationship with typical emotion regulation strategy in young adulthood

**Figure 4.**
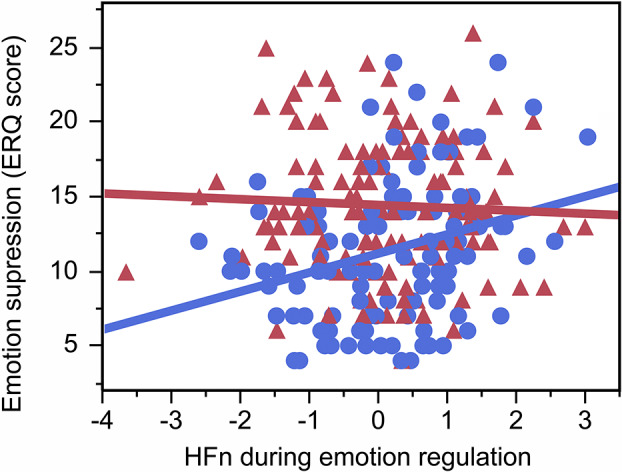
Greater HFn during emotion regulation was associated with more emotion suppression in women (blue; *R*^2^ = 0.08, beta = 0.29, *p* = 0.002). Women are depicted in blue, men are depicted in red.

Greater HFn during emotion regulation interacted with sex and predicted more emotion suppression (beta = −0.15, FDRp = 0.01) in young adulthood. Posthoc analyses revealed that the relationship between greater HFn during emotion regulation and more emotion suppression was significant in women (R^2^ = 0.08, beta = 0.29, p = 0.002; Figure 4) but not men (beta = −0.02, p = 0.83). There was no similar interaction between HFn and sex on emotion reappraisal (beta = 0.03, FDRp = 0.45) or a main effect of HFn on reappraisal (beta = 0.09, p = 0.16).

### Participants’ feelings during the observe versus regulate conditions of the fMRI task

As indicated in the graph provided in Supplementary Figure 4 and in the statistics overview table provided in Supplementary Table 7, the regulate condition was associated with significantly more „not at all “answers (z = 4.83, p < 0.001) than the observe condition, indicating that the emotion regulation helped participants not to feel uncomfortable. Consistently, the observe condition was associated with significantly more „quite a bit “(z = −3.35, p = 0.001) and „very much “(z = −2.79, p = 0.005) answers than the regulate condition, indicating that simple observation of the negative images made the participants feel uncomfortable.

## Discussion

We have conducted a neuroimaging follow-up of a prenatal birth cohort and studied the relationships between maternal depression during the perinatal period and early life of the offspring and brain response during an emotion regulation task in young adulthood. We also assessed whether these relationships might be reflected by typical emotion regulation strategy (emotion suppression versus emotion reappraisal) in young adulthood and particularly whether the altered brain response might explain the relationship between maternal perinatal depression and the typical emotion regulation strategy in the young adult offspring. Moreover, we also tested whether the altered brain response might be reflected by altered skin conductance and heart rate variability during the emotion regulation task and whether the altered skin conductance and heart rate variability could predict the typical emotion regulation strategy.

We showed that maternal depression 2 weeks after birth (above and beyond maternal depression experienced at other times during the perinatal period and early life of the offspring) predicted greater response in the ventromedial prefrontal region including superior frontal gyrus and anterior orbitofrontal cortex during emotion regulation in young adult women but not men. Moreover, this altered brain response during emotion regulation in young adult women was associated with greater habitual use of emotion suppression, which is typically a maladaptive emotion regulation strategy. In addition, the increased brain response in the right frontal cluster mediated the relationship between maternal depression 2 weeks after birth and emotion suppression in the young adult daughters. We speculate that the greater brain response in the frontal regions might reflect greater inefficiency of the prefrontal circuitry during emotion regulation and that young adult women, whose mothers suffered from more depressive symptoms 2 weeks after birth, had to make more cognitive effort to regulate their negative affect. These findings also suggest that women are more vulnerable to the long-lasting changes of early adversity and might also, at least in part, explain the greater prevalence of depression in women.

These findings substantially extend the research of Maruyama et al (Maruyama et al., 2023), who linked maternal depression during the offspring’s childhood to worse emotion regulation skills in adolescence, and the research of Li et al (Li et al., 2019), who linked parental emotion dysregulation in offspring’s childhood to worse emotion regulation in childhood. They point out the importance of timing of the exposure and that the early postpartum period (e.g 2 weeks after birth) might be particularly important not only for mother–infant attachment (Śliwerski et al., 2020), but also for the development of emotion regulation skills in the offspring. The sex-specificity of our findings then supports the research of Nolen-Hoeksema (Nolen-Hoeksema, 2012) and McRae et al (McRae et al., 2008), who reported sex differences in emotion regulation and brain response during emotion regulation.

The relationship between maternal depression during pregnancy (above and beyond maternal depression experienced at other times during the perinatal period and early life of the offspring) and greater brain response in the occipital cluster in women might be related to altered sensory development in utero. However, activity in this cluster was not further associated with altered skin conductance, heart rate variability, or the typical emotion regulation strategy. These findings are suggestive of a temporal specificity, whereby maternal depression 2 weeks after birth but not during pregnancy particularly affects emotion regulation skills in young adulthood. Based on this time-specificity, we speculate that the relationship between maternal depression 2 weeks after birth and worse emotion regulation skills in young adulthood might not stem from a direct effect of altered glucocorticoids or maternal immune activation during the prenatal development but may instead be mediated by socio-emotional developmental factors such as an insecure attachment or an indirect effect on cortisol levels in young adulthood. In fact, it has been demonstrated that cortisol might exert beneficial effects on the cognitive downregulation of affect elicited by intensive negative pictures (Jentsch et al., 2019; Langer et al., 2022).

We also demonstrated that the altered brain response during emotion regulation in women was further associated with skin conductance and heart rate variability during emotion regulation and that this altered skin conductance and heart rate variability predicted the typical emotion regulation strategy. These findings suggest that exposure to maternal depression may impact emotion regulation on the neural and physiological levels, which may in turn translate into broader patterns of habitual use of adaptive versus maladaptive emotion regulation skills. The fact that we found associations between greater emotion reappraisal, the more adaptive emotion regulation strategy, and greater skin conductance latency as well as greater nSCR is consistent with previous research that found lower skin conductance among depressed patients versus healthy controls. (Kim et al., 2019; Ward et al., 1983). The relationship between greater HFn, an index of parasympathetic modulation, and greater emotion suppression in women is similarly consistent with An et al (An et al., 2020), who reported an association between greater HFn and increased risk of depression. The concurrent acquisition of skin conductance, heart rate variability and brain response during fMRI, as done in our current study, is rare, mostly due to the difficult pre-processing of the physiology data and thus our study provides important evidence that the trough to peak (TTP) analysis is well suited for the analysis of skin conductance response during fMRI task and that the altered emotion regulation manifests particularly in the nSCR, skin conductance latency, and HFn.

Our findings are limited by the design of the current study and do not allow us to make any conclusions about the relationship between maternal depression occurring before or after these 4 sensitive periods and emotion regulation skills in the offspring or about the relationship between emotion regulation skills in the mother and the young adult offspring. We also did not assess the genetics of the mother and the offspring and thus could not test to what extent the observed time-specific relationships between maternal depression 2 weeks after birth and the brain function and physiology during the emotion regulation task as well as the emotion regulation skills in the offspring might be mediated by genes. Still, the current study has a number of methodological strengths including (1) prospective longitudinal study design using data from a prenatal birth cohort with detailed information on maternal depression during the perinatal period and early life of the offspring assessed at four different timepoints throughout the pregnancy and after birth, (2) assessment of emotion regulation skills in young adulthood using both fMRI emotion regulation task as well as behavioral data regarding the typical emotion regulation strategy, (3) combination of the brain response with skin conductance and heart rate variability data collected during the fMRI task, and (4) even distribution of sex within the sample, enabling us to investigate the role of sex in these relationships. Therefore, our findings regarding the time-specific relationship between maternal depression during the perinatal period and brain function during an emotion regulation task as well as the typical emotion regulation strategy in young adulthood provide unique insight into the developmental programming of mental health.

## Supporting information

Mareckova et al. supplementary material Mareckova et al. supplementary material

## Data Availability

Data are available from the first author upon reasonable request.

## Supplementary material

The supplementary material for this article can be found at http://doi.org/10.1017/S0033291725000042.
