# Supplementary material for: Maternal depression during the perinatal period and its relationship with emotion regulation in young adulthood: An fMRI study in a prenatal birth cohort
Source: Psychol Med. 2025 Feb 12;55:e39. doi: 10.1017/S0033291725000042 (PMC12017355; doi:10.1017/S0033291725000042)

**SUPPLEMENTARY MATERIALS**

**Supplementary Table 1: Demographics table**

**Supplementary Table 2: Maternal depression during the perinatal period.**

| **EPDS measurement** | **Mean** | **SD** | **Range** |
| --- | --- | --- | --- |
| **Mid-pregnancy** | 11.74 | 2.49 | 6 - 23 |
| **2 weeks after birth** | 6.43 | 4.15 | 0 - 20 |
| **6 months after birth** | 5.67 | 4.24 | 0 - 17 |
| **18 months after birth** | 5.92 | 4.91 | 0 - 24 |

|  | **Whole sample**  **(n=163)** | **Women**  (n=80) | **Men**  (n=83) | **Sex difference** |
| --- | --- | --- | --- | --- |
| **Sex (**%) |  | 49% | 51% |  |
| **Ethnicity**  % White Caucasian | 100% | 100% | 100% | No difference. |
| **Age at MRI** in years (M, SD) | M=28.66, SD=0.56 | M=28.68, SD=0.57 | M=28.64, SD=0.55 | No difference. |
| **BMI at MRI** (M, SD) | M=24.12, SD=4.05 | M=24.23, SD=4.18 | M=24.02, SD=3.94 | No difference. |
| **Education**  % completed elementary school  % completed high school  % completed an undergraduate degree  % completed a master's degree  % completed PhD | 0.61%  24.54%  10.43%  63.19%  1.23% | 1.25%  23.75%  8.75%  66.25%  0% | 0%  25.30%  12.05%  60.24%  2.41% | No difference. |
| **ERQ score Reappraisal** (M, SD) | M=29.40, SD=6.07 | M=29.48, SD=6.10 | M=29.34, SD=6.08 | No difference. |
| **ERQ score Suppression** (M, SD) | M=13.15, SD=4.99 | M=12.71, SD=4.79 | M=13.58, SD=5.17 | No difference. |
| **Maternal Education**  % missing data  % not completed high school  % completed high school  % completed university  % completed postgraduate educ. | 19.20%  0%  49.00%  30.00%  1.80% | 15.00%  0%  52.5%  30.00%  2.50% | 25.3%  0%  46.9%  26.6%  1.2% | No difference. |

**Supplementary Table 3:** Cronbach alphas for the variables regarding maternal perinatal depression and the emotion regulation skills in the offspring.

| **Variable** | **Cronbach’s alpha** |
| --- | --- |
| Maternal depression in mid-pregnancy | 0,82 |
| Maternal depression 2 weeks after birth | 0,83 |
| Maternal depression 6 months after birth | 0,83 |
| Maternal depression 18 months after birth | 0,85 |
| Emotion regulation - Reappraisal | 0,73 |
| Emotion regulation - Suppression | 0,82 |

**Supplementary Table 4** – Pairwise correlations among the 4 maternal depression variables and emotion regulation strategy in the offspring.

| **Variable** | **by Variable** | **Correlation** | **P-value** |
| --- | --- | --- | --- |
| Maternal depression 2 weeks after birth | Maternal depression in mid-pregnancy | 0.4 | < 0.0001 |
| Maternal depression 6 months after birth | Maternal depression in mid-pregnancy | 0.26 | 0.0007 |
| Maternal depression 6 months after birth | Maternal depression 2 weeks after birth | 0.59 | < 0.0001 |
| Maternal depression 18 months after birth | Maternal depression in mid-pregnancy | 0.32 | < 0.0001 |
| Maternal depression 18 months after birth | Maternal depression 2 weeks after birth | 0.5 | < 0.0001 |
| Maternal depression 18 months after birth | Maternal depression 6 months after birth | 0.53 | < 0.0001 |
| Emotion regulation - Reappraisal | Maternal depression in mid-pregnancy | -0.04 | 0.5726 |
| Emotion regulation - Reappraisal | Maternal depression 2 weeks after birth | 0.02 | 0.7594 |
| Emotion regulation - Reappraisal | Maternal depression 6 months after birth | -0.03 | 0.7357 |
| Emotion regulation - Reappraisal | Maternal depression 18 months after birth | -0.14 | 0.0813 |
| Emotion regulation - Suppression | Maternal depression in mid-pregnancy | 0.13 | 0.0906 |
| Emotion regulation - Suppression | Maternal depression 2 weeks after birth | 0.03 | 0.7358 |
| Emotion regulation - Suppression | Maternal depression 6 months after birth | 0.03 | 0.6633 |
| Emotion regulation - Suppression | Maternal depression 18 months after birth | 0.04 | 0.5958 |
| Emotion regulation - Suppression | Emotion regulation - Suppression | 0.05 | 0.5528 |

**Supplementary Table 5:** Brain response during the observe negative vs. neutral contrast (FWEp<0.05).

| # cluster | cluster - level | | | voxel - level | | x,y,z | | | AAL Brain Region |
| --- | --- | --- | --- | --- | --- | --- | --- | --- | --- |
|  | FWE p | # voxels | | FWE p | T |  |  |  |  |
| 1 | <0.001 | 290 | | <0.001 | 9.67 | 51 | -70 | 2 | 237 Temporal_Mid_R (aal); 33 BA 37; 31 BA 39; 22 BA 19; 18 Occipital_Inf_R; 18 Temporal_Inf_R (aal); 16 BA 22; 13 Occipital_Mid_R (aal); 2 Temporal_Sup_R (aal); 2 BA 21 |
| 2 | <0.001 | 207 | | <0.001 | 8.99 | -51 | -73 | 5 | 96 Temporal_Mid_L (aal); 58 Occipital_Mid_L (aal); 35 BA 37; 28 Occipital_Inf_L (aal); 20 BA 39; 20 BA 19; 5 BA 22; 2 BA 21 |
| 3 | <0.001 | 313 | | <0.001 | 7.93 | 45 | -49 | 50 | 200 Parietal_Inf_R (aal); 137 BA 40; 41 SupraMarginal_R (aal); 31 Angular_R (aal); 28 Parietal_Sup_R (aal); 2 BA 7; 1 BA 2 |
| 4 | <0.001 | 398 | | <0.001 | 7.86 | -33 | -49 | 41 | 280 Parietal_Inf_L (aal); 137 BA 40; 51 Postcentral_L (aal); 29 Parietal_Sup_L (aal); 22 BA 2; 7 SupraMarginal_L (aal); 4 Angular_L (aal); 2 BA 5; 2 BA 7; 2 BA 3 |
| 5 | <0.001 | 153 | | <0.001 | 7.70 | -6 | 23 | 38 | 45 Frontal_Sup_Medial_L (aal); 37 BA 32; 37 Supp_Motor_Area_L (aal); 20 BA 8; 19 Frontal_Sup_Medial_R (aal); 19 Supp_Motor_Area_R (aal); 17 Cingulum_Mid_R (aal); 11 Cingulum_Mid_L (aal); 11 BA 6; 9 BA 9; 5 Cingulum_Ant_L (aal) |
| 6 | <0.001 | 214 | | <0.001 | 7.36 | -6 | -67 | 41 | 106 Precuneus_R (aal); 100 Precuneus_L (aal); 90 BA 7; 9 Superior Parietal Lobule; 4 Cuneus_R (aal); 2 Occipital_Sup_L (aal); 2 Parietal_Sup_L (aal) |
| 7 | <0.001 | 15 | | <0.001 | 6.40 | -30 | -58 | -31 | 9 Cerebelum_6_L (aal); 6 Cerebelum_Crus1_L (aal); 4 Declive; 1 Uvula; 1 Tuber |
| 8 | <0.001 | 25 | | <0.001 | 6.35 | 42 | 17 | -1 | 12 Insula_R (aal); 7 Frontal_Inf_Oper_R (aal); 6 BA 47; 5 Frontal_Inf_Orb_R (aal); 1 Frontal_Inf_Tri_R (aal) |
| 9 | <0.001 | | 11 | 0.001 | 5.79 | -42 | 32 | 29 | 11 Frontal_Mid_L (aal); 1 BA 9 |
| 10 | <0.001 | | 16 | 0.001 | 5.79 | -36 | 17 | 2 | 14 Insula_L (aal); 4 BA 47; 2 Frontal_Inf_Orb_L (aal); 2 BA 13 |
| 11 | <0.001 | | 21 | 0.001 | 5.68 | 42 | 50 | 8 | 21 Frontal_Mid_R (aal); 5 BA 10; 1 BA 46 |
| 12 | <0.001 | | 13 | 0.002 | 5.59 | 0 | -28 | 26 | 5 Cingulum_Mid_R (aal); 4 BA 23 |
| 13 | <0.001 | | 7 | 0.005 | 5.42 | 33 | 26 | 2 | 6 Insula_R (aal); 2 BA 45; 1 BA 47; 1 BA 13 |
| 14 | <0.001 | | 11 | 0.005 | 5.41 | 45 | 38 | 23 | 9 Frontal_Mid_R (aal); 2 Frontal_Inf_Tri_R (aal); 1 BA 10; 1 BA 9 |

**Supplementary Table 6:** Brain response during the regulate negative vs. observe negative contrast (FWEp<0.05).

| # cluster | cluster - level | | voxel - level | | x,y,z | | | AAL Brain Region |
| --- | --- | --- | --- | --- | --- | --- | --- | --- |
|  | FWE p | # voxels | FWE p | T |  |  |  |  |
| 1 | <0.001 | 7280 | <0.001 | 15.29 | -54 | -40 | 2 | 747 Temporal_Mid_L (aal); 725 Frontal_Mid_L (aal); 488 BA 6; 461 Frontal_Inf_Tri_L (aal); 343 Supp_Motor_Area_L (aal); 315 Frontal_Mid_R (aal); 266 Supp_Motor_Area_R (aal); 259 BA 9; 236 Frontal_Inf_Orb_L (aal); 236 Frontal_Sup_L (aal); 232 BA 21; 230 Parietal_Inf_L (aal); 214 BA 10; 212 BA 38; 211 Precentral_L (aal); 201 Frontal_Sup_Medial_L (aal); 198 BA 8; 193 BA 40; 189 Angular_L (aal); 181 Temporal_Pole_Sup_L (aal); 166 BA 47; 160 Frontal_Inf_Tri_R (aal); 157 Frontal_Sup_R (aal); 151 Frontal_Inf_Oper_L (aal); 147 Frontal_Inf_Orb_R (aal); 145 BA 22; 139 BA 45; 130 SupraMarginal_L (aal); 126 Temporal_Pole_Mid_R (aal); 125 Precentral_R (aal); 124 Temporal_Pole_Sup_R (aal); 124 Temporal_Sup_L (aal); 122 Frontal_Inf_Oper_R (aal); 113 Insula_R (aal); 102 Frontal_Sup_Medial_R (aal); 95 Temporal_Pole_Mid_L (aal); 91 Temporal_Inf_R (aal); 73 Cingulum_Mid_R (aal); 72 Temporal_Inf_L (aal); 68 BA 20; 67 Insula_L (aal); 64 BA 46; 61 Rolandic_Oper_R (aal); 60 BA 44; 58 Frontal_Mid_Orb_L (aal); 58 BA 32; 52 BA 13; 40 BA 39; 28 Cingulum_Mid_L (aal); 18 BA 7; 18 Temporal_Mid_R (aal); 16 BA 11; 14 Rolandic_Oper_L (aal); 13 Parietal_Sup_L (aal); 10 Postcentral_L (aal); 6 BA 4; 5 Frontal_Mid_Orb_R (aal); 4 BA 24; 3 Temporal_Sup_R (aal); 2 Putamen_R (aal); 2 BA 42; 2 Cingulum_Ant_L (aal); 1 Cingulum_Ant_R (aal); 1 Frontal_Sup_Orb_L (aal) |
| 2 | <0.001 | 796 | <0.001 | 10.51 | 48 | -31 | -4 | 251 Temporal_Mid_R (aal); 203 SupraMarginal_R (aal); 140 BA 40; 133 Temporal_Sup_R (aal); 103 Angular_R (aal); 73 Parietal_Inf_R (aal); 66 BA 22; 41 BA 21; 7 BA 13; 5 BA 39; 2 BA 2; 1 BA 41 |
| 3 | <0.001 | 995 | <0.001 | 9.86 | 18 | -82 | -34 | 243 Cerebelum_Crus1_R (aal); 176 Cerebelum_6_R (aal); 175 Cerebelum_Crus2_R (aal); 136 Lingual_R (aal); 98 Occipital_Inf_R (aal); 95 Uvula; 85 Fusiform_R (aal); 75 Tuber; 74 BA 18; 73 Pyramis; 54 Calcarine_R (aal); 42 Cuneus; 37 Culmen; 27 BA 19; 23 BA 17; 12 Occipital_Mid_R (aal); 5 Vermis_7 (aal); 4 BA 37; 4 Cerebelum_7b_R (aal); 1 Vermis_6 (aal); 1 Occipital_Sup_R (aal) |
| 4 | <0.001 | 891 | <0.001 | 9.44 | -24 | -97 | -4 | 213 Occipital_Mid_L (aal); 154 Cerebelum_Crus1_L (aal); 145 Declive; 135 Fusiform_L (aal); 115 Occipital_Inf_L (aal); 99 Cerebelum_6_L (aal); 85 BA 18; 75 Inferior Occipital Gyrus; 70 Lingual_L (aal); 67 BA 19; 67 Cuneus; 46 Culmen; 45 Tuber; 44 Cerebelum_Crus2_L (aal); 29 BA 17; 25 Calcarine_L (aal); 23 Uvula; 15 Pyramis; 12 BA 37; 7 Temporal_Inf_L (aal); 3 Occipital_Sup_L (aal) |
| 5 | <0.001 | 68 | <0.001 | 8.28 | -12 | 8 | 11 | 52 Caudate_L (aal); 1 Putamen_L (aal) |
| 6 | <0.001 | 7280 | <0.001 | 7.64 | -3 | -49 | 50 | 198 Precuneus_L (aal); 74 BA 7; 46 Parietal_Sup_L (aal); 13 BA 31; 6 Precuneus_R (aal); 3 Cingulum_Mid_L (aal); 1 Cuneus_L (aal) |
| 7 | <0.001 | 796 | <0.001 | 7.52 | 27 | -67 | -55 | 74 Cerebelum_8_R (aal); 15 Cerebelum_7b_R (aal); 5 Cerebelum_Crus2_R (aal) |
| 8 | <0.001 | 995 | <0.001 | 6.99 | 9 | 5 | 8 | 83 Caudate_R (aal); 11 Putamen_R (aal) |
| 9 | <0.001 | 891 | <0.001 | 6.44 | -3 | 65 | -19 | 13 BA 11; 10 Frontal_Sup_Orb_R (aal); 8 Rectus_L (aal) |
| 10 | <0.001 | 68 | <0.001 | 6.02 | -39 | -55 | -46 | 24 Cerebelum_8_L (aal); 7 Cerebelum_7b_L (aal); 2 Cerebelum_Crus2_L (aal) |
| 11 | <0.001 | 266 | <0.001 | 5.93 | -21 | -1 | 5 | 3 Putamen_L (aal); 3 Pallidum_L (aal) |
| 12 | 0.001 | 94 | <0.001 | 5.91 | 39 | -88 | 14 | 5 Occipital_Mid_R (aal) |
| 13 | <0.001 | 110 | 0.001 | 5.74 | -21 | 68 | 8 | 6 Frontal_Sup_L (aal); 5 BA 10 |
| 14 | <0.001 | 37 | 0.001 | 5.66 | 6 | -22 | -4 | 5 Midbrain; 1 Thalamus_R (aal) |
| 15 | <0.001 | 33 | 0.003 | 5.52 | -21 | 14 | -4 | 5 Putamen_L (aal) |
| 16 | <0.001 | 6 | 0.005 | 5.42 | -24 | -88 | 32 | 12 Occipital_Sup_L (aal); 6 BA 19; 1 BA 7 |
| 17 | <0.001 | 5 | 0.006 | 5.39 | 6 | -19 | 41 | 4 BA 24; 3 Cingulum_Mid_L (aal) |

**Supplementary Table 7:** Statistical analyses of the ratings regarding participants‘ feelings during the observe vs. regulate conditions of the fMRI task. Median values are followed by lower/upper quartile values in the brackets.

| rating | observe | regulate | regulate-observe | Z-value | p-value |
| --- | --- | --- | --- | --- | --- |
| not at all | 0.191  (0.06; 0.49) | 0.273  (0.09; 0.62) | 0.041  (-0.02; 0.12) | 4.83 | <0.001 |
| a little | 0.333  (0.23; 0.50) | 0.350  (0.21; 0.52) | 0.000  (-0.09; 0.09) | -0.24 | 0.810 |
| quite a bit | 0.231  (0.05; 0.36) | 0.182  (0.05; 0.33) | 0.000  (-0.09; 0.04) | -3.35 | 0.001 |
| very much | 0.046  (0.00; 0.18) | 0.046  (0.00; 0.10) | 0.000  (-0.04; 0.00) | -2.79 | 0.005 |

**Supplementary Figure 1:** **The amount of movement during scanning.** There was no difference in mean framewise displacement between men and women (Man-Whitney test, p<0.05).


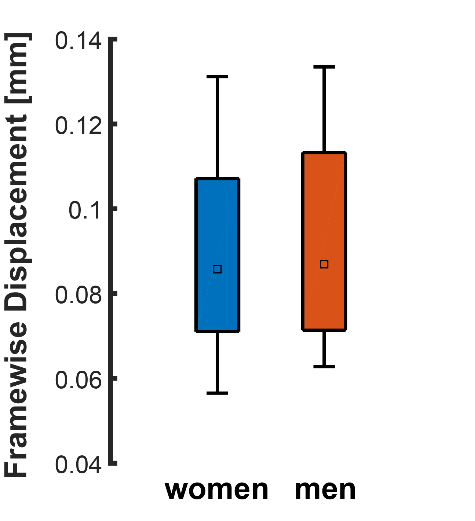


**Supplementary Figure 2: Brain response during the observe negative vs. neutral contrast** (FWEp<0.05).

**
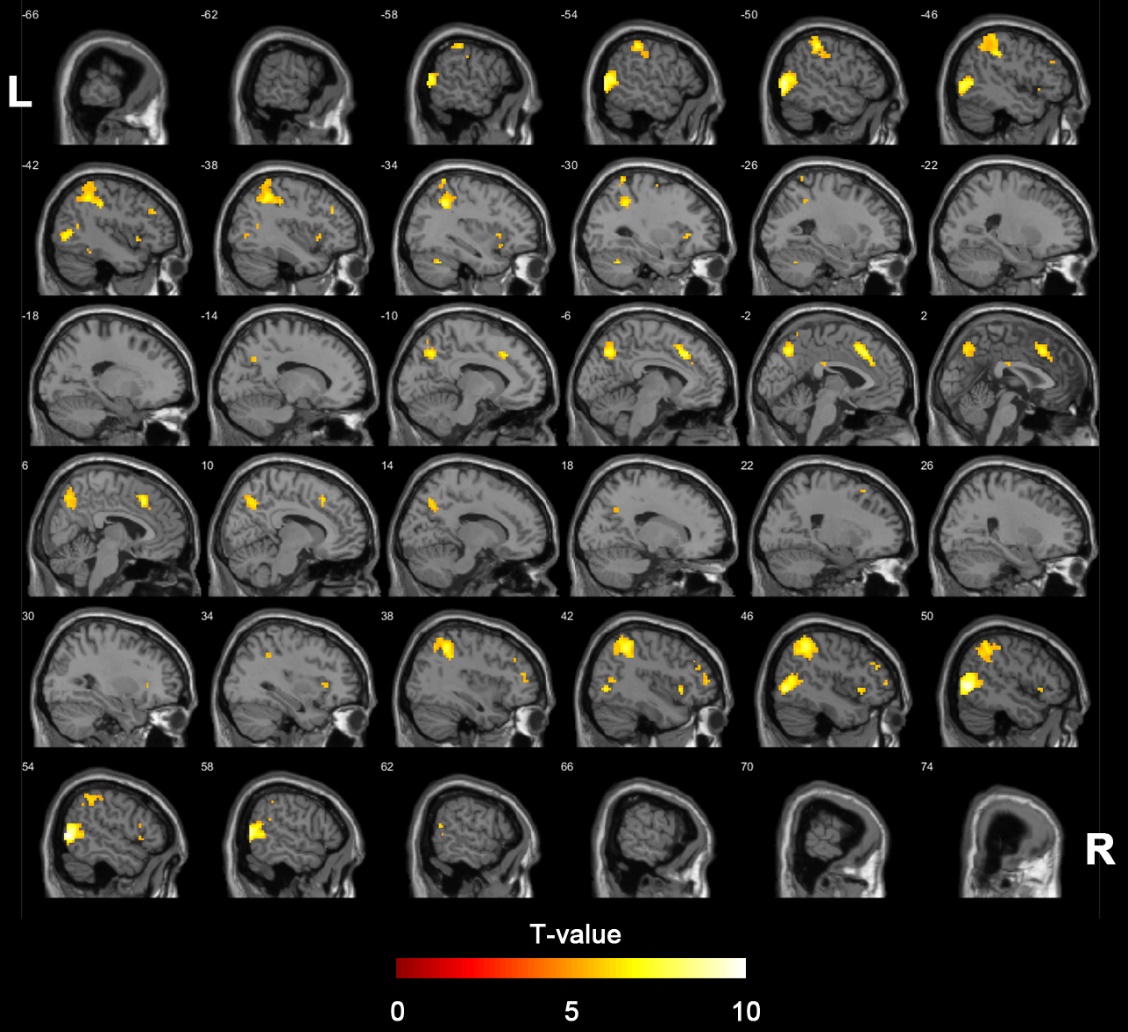
**

**Supplementary Figure 3: Brain response during the regulate negative vs. observe negative contrast** (FWEp<0.05).


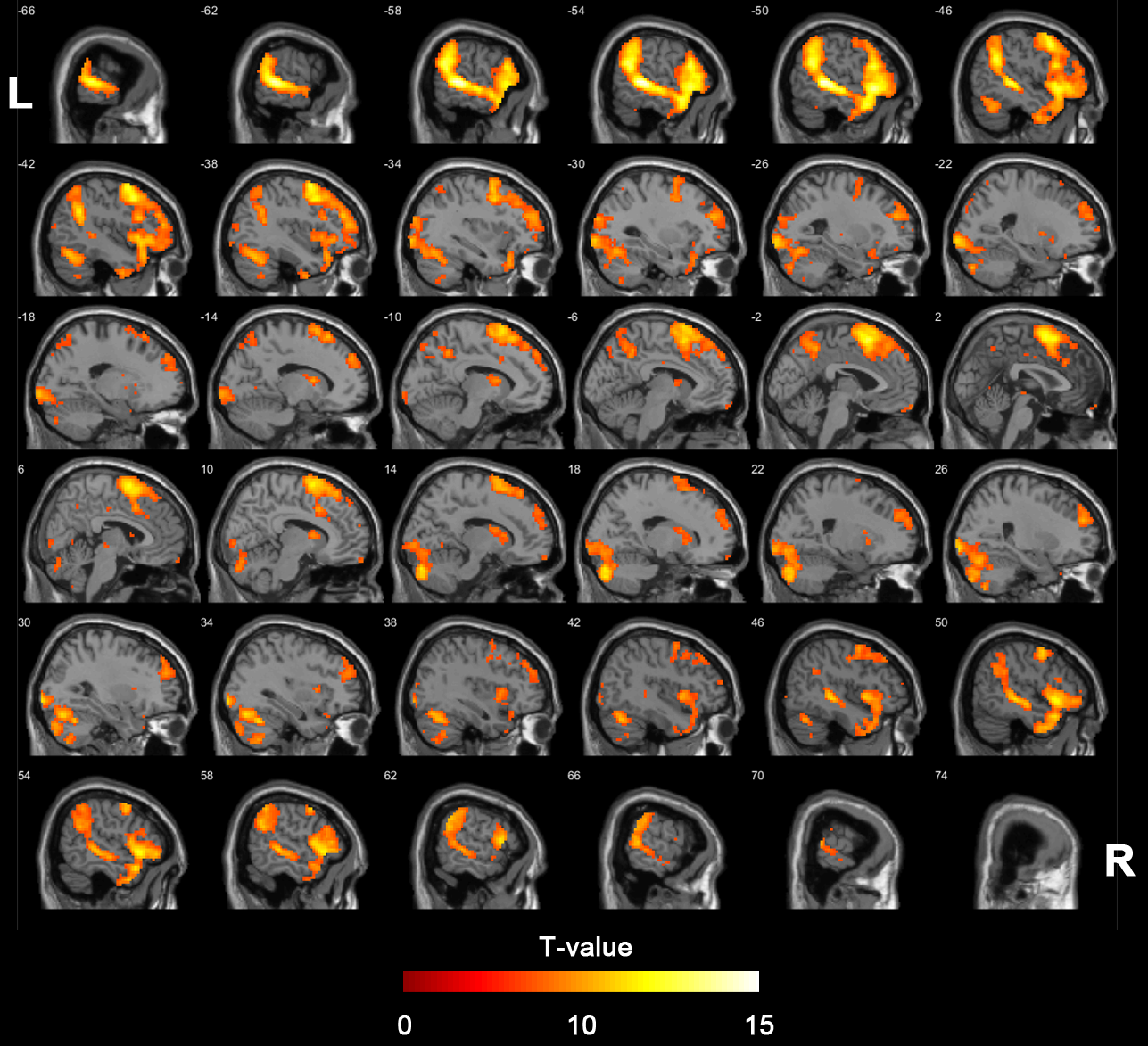


**Supplementary Figure 4:** **Rating of participants‘ feelings during the fMRI task.** During each trial, participants rated „How uncomfortable do you feel right now?” using one of the following answers: “not at all – a little – quite a bit – very much”. The regulate condition (green) was associated with significantly more „not at all“ answers (z=4.83, p<0.001) than the observe condition (grey), indicating that the emotion regulation helped participants not to feel uncomfortable. Consistently, the observe condition (grey) was associated with significantly more „quite a bit“ (z=-3.35, p=0.001) and „very much“ (z=-2.79, p=0.005) answers than the regulate condition (green), indicating that simple observation of the negative images made the participants feel uncomfortable. The box plots show median, quartiles and lowest/highest deciles over all subjects. The gray/green boxes show observe/regulate condtions, respectively.


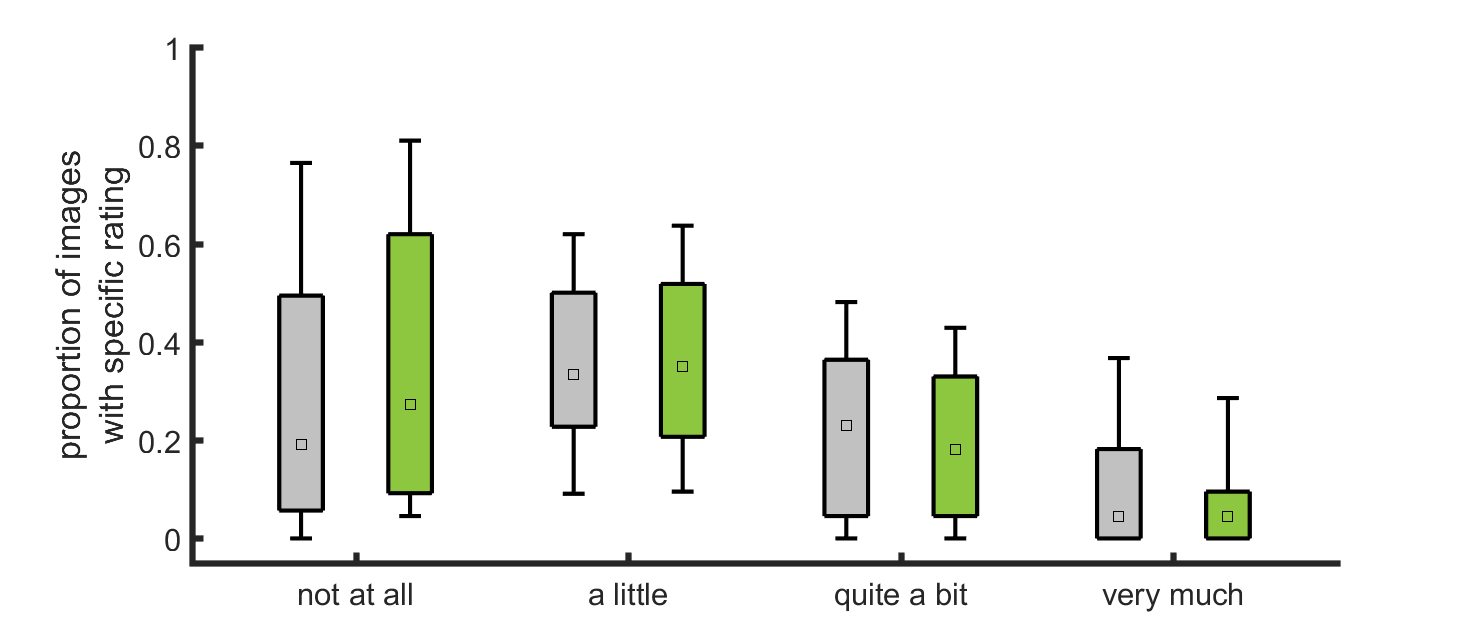

Supplement: Mareckova et al. supplementary material [file S0033291725000042sup001.docx]
